# Integration of High-Brightness QLED-Excited Diamond Magnetic Sensor

**DOI:** 10.3390/mi16091021

**Published:** 2025-09-04

**Authors:** Pengfei Zhao, Junjun Du, Jinyu Tai, Zhaoqi Shang, Xia Yuan, Yuanyuan Shi

**Affiliations:** 1 School of Mechanical Engineering, North University of China, Taiyuan 030051, China; wings215@yeah.net (P.Z.); shangzhaoqi1@163.com (Z.S.); 20060201@nuc.edu.cn (Y.S.); 2 Shandong Huaguang Optoelectronic Co., Ltd., Jinan 250000, China; dujj@inspur.com; 3 State Key Laboratory of Dynamic Measurement Technology, Shanxi Province Key Laboratory of Quantum Sensing and Precision Measurement, North University of China, Taiyuan 030051, China; tjy15735113671@163.com

**Keywords:** NV center, QLED, magnetic sensing array, magnetic field detection

## Abstract

The nitrogen-vacancy (NV) center magnetic sensor, leveraging nitrogen-vacancy quantum effects, enables high-sensitivity magnetic field detection via optically detected magnetic resonance (ODMR). However, conventional single-point integrated devices suffer from limitations such as inefficient regional magnetic field detection and challenges in discerning the directional variations of dynamic magnetic fields. To address these issues, this study proposes an array- based architecture that innovatively substitutes the conventional 532 nm laser with quantum-dot light-emitting diodes (QLEDs). Capitalizing on the advantages of QLEDs—including compatibility with micro/nano-fabrication processes, wavelength tunability, and high luminance—a 2 × 2 monolithically integrated magnetometer array was developed. Each sensor unit achieves a magnetic sensitivity of below 26 nT·Hz^−1/2^ and a measurable range of ±120 μT within the 1–10 Hz effective bandwidth. Experimental validation confirms the array’s ability to simultaneously resolve multi-regional magnetic fields and track dynamic field orientations while maintaining exceptional device uniformity. This advancement establishes a scalable framework for the design of large-scale magnetic sensing arrays, demonstrating significant potential for applications requiring spatially resolved and directionally sensitive magnetometry.

## 1. Introduction

The diamond NV center magnetic sensor is an advanced magnetic sensing technology based on the quantum effects of nitrogen-vacancy (NV) color centers [1,2,3]. When diamond is irradiated with 532 nm green light, the electrons in the ground state of the diamond NV color center are efficiently excited to the excited state. Subsequently, some of the electrons in the excited state decay to the ground state through radiative relaxation, emitting red fluorescence with a wavelength range of 600–800 nm. Simultaneously applying microwave signals to regulate the electron spin of NV color centers, a resonant transition occurs to generate ODMR spectra, representing the spin-dependent fluorescence response of diamond NV color centers under optical excitation and microwave manipulation [4,5]. Through Optically Detected Magnetic Resonance (ODMR) technology, NV center magnetometers can capture weak magnetic field signals. With core technical advantages such as ultra-high sensitivity, high spatial resolution, and vector measurement capability, NV center magnetometers demonstrate strong application potential in fields including biomedicine, materials science, and industrial inspection [6,7,8,9,10,11]. Existing commercial magnetic sensors mainly include Hall elements, xMR devices, and optical pump magnetometers, among which magnetoresistive sensors have made significant progress in low-frequency noise (such as the TDK Nivio xMR). Magnetoresistive devices rely on changes in the relative angle between the free layer and reference layer and are highly sensitivity to magnetic field changes, but there are issues with repeatability and stability [12]. However, a diamond NV center can measure the absolute amount of magnetic field through spin-level splitting, and there are no issues such as hysteresis or zero temperature drift. At present, the sensitivity of diamond NV magnetic sensors has reached the fT level [13], but the laser volume is too large, the cost is too high, and integration is low. Therefore, this article innovatively studies the excitation technology of quantum-dot light-emitting diodes (QLEDs), aiming to achieve the integration of diamond NV magnetometer arrays.

Presently, research on solid-state spin magnetometers based on diamond nitrogen-vacancy (NV) centers primarily focuses on the integration of single magnetometer units [14,15,16,17]. While integrated NV center magnetometers can detect magnetic field variations at individual points, their capability is severely limited for spatially distributed magnetic fields (regional magnetic fields). To map such fields, the magnetometers must operate in a scanning mode, which significantly reduces detection efficiency. Furthermore, for dynamically varying magnetic fields (dynamic magnetic fields), integrated magnetometers can only quantify field magnitude but fail to determine the direction of magnetic field variations [18,19]. To overcome these challenges, transitioning from single-device integration to array-based configurations is essential [20].

To achieve array compatibility in diamond NV center magnetometers, comprehensive array-oriented engineering of all functional components is required. In current research, photodetectors, microwave antennas, and diamond substrates can be systematically arrayed using micro/nano-fabrication processes. However, the miniaturization and array integration of conventional 532 nm laser systems pose significant technical hurdles, necessitating an alternative excitation light source [21,22]. Here, we propose quantum-dot light-emitting diodes (QLEDs) as a transformative solution for NV center excitation. QLEDs exhibit high brightness, narrow full width at half maximum, and tunable emission wavelengths, ensuring optimal spectral alignment with NV center optical transitions [23,24,25]. Crucially, QLED devices enable large-scale integration through compatibility with micro-nanofabrication techniques, while their individually addressable pixel architecture permits precise control over illumination patterns for high-density array configurations [26,27,28].

In this study, we utilized the controllable illumination areas of QLEDs to propose a monolithically integrated, arrayed NV color-center magnetometer. For preliminary validation, we constructed a 2 × 2 NV color-center magnetometer array model on the same QLED substrate: four magnetometer units were built within four independent illumination zones, and the consistency of these devices was tested. Through simulated experiments, we demonstrated that the magnetometer array can detect magnetic fields in different regions and track dynamic magnetic fields. This provides a novel solution for the future development of large-scale arrayed magnetometers. Compared with classical physics based high-sensitivity magnetic sensors, although there is a certain gap in sensitivity, the innovative use of QLED excitation technology has achieved the integration of NV magnetometer arrays, providing a new paradigm for magnetic field analysis in quantum sensing [29,30].

## 2. Materials and Methods

### 2.1. Materials and Preparation of QLED

In the fabrication of QLEDs, the materials utilized include commercially available quantum dots (QDs, CdSe/ZnS core-shell), poly(3,4-ethylenedioxy-thiophene)-poly(styrenesul-fonate)(PEDOT:PSS), poly(9,9-dioctylfluorene-co-N-(4-(3-methylpropyl))diphenylamine)(TFB), and zinc magnesium oxide (ZnMgO). All materials were sourced from Suzhou XingShuo Nanotech Co., Ltd. (Suzhou, China). All chemical reagents were used as received, without any purification prior to their application in the fabrication process.

### 2.2. Preparation of QLED-Excited Diamond Magnetic Sensor

**Figure 1:**
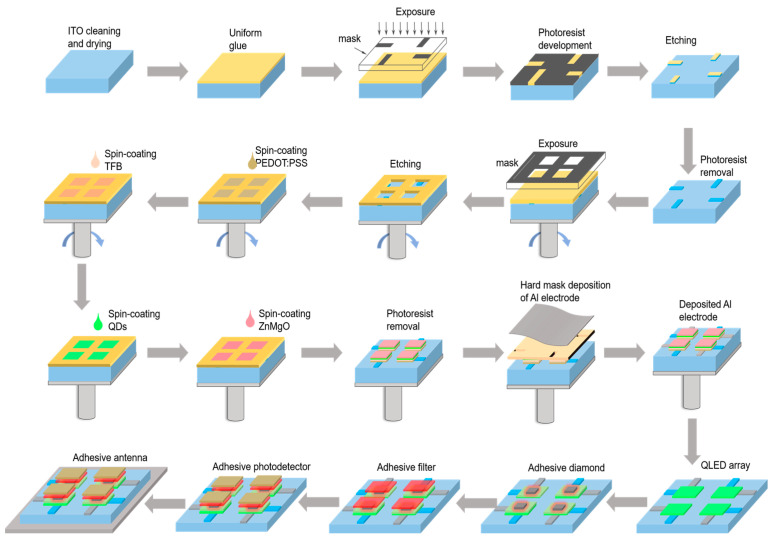
Schematic diagram of QLED-NV preparation process.

QLED devices were prepared by the mature spin-coating method as shown in Figure 1. ITO film with lower resistivity (6–8 Ω/sq) is selected as the anode substrate. Then, the ITO film substrate is ultrasonically cleaned with acetone, isopropanol, methanol, and deionized water and patterned on the film substrate using photolithography. Then, PEDOT:PSS, TFB, QDs, and ZnMgO are spin-coated and deposited at a speed of 1500 r/min, using a graphical mask to achieve evaporation treatment of patterned aluminum electrodes. Finally, QLED devices are combined with photodetectors and filtered, and diamond and antennae are encapsulated in a glove box using UV-curable resin (UV glue) for subsequent characterization and analysis.

### 2.3. Characterization of QLEDs

We evaluated the relevant performance of the QLEDs under ambient conditions. The electroluminescence (EL) spectrum of the QLED was measured using an Ocean Optics (Shanghai, China) QE65000 spectrometer and a Xi’an Lik Optoelectronic Technology Co., Ltd. (Xi’an, China) Keysight B2902A digital source meter. The current density-voltage (J-V) characteristics of the QLED were assessed using a Keysight B2902A digital source meter from Xi’an Lik Optoelectronic Technology Co., Ltd., Ltd (Xi’an, China). Additionally, the luminance of the QLED was measured with a Photo Research PR-655 QLED spectroradiometer from Pioneer Technology (Hong Kong) Co., Ltd (Hong Kong, China).

### 2.4. Characterization of the Magnetometer Array

**Figure 2:**
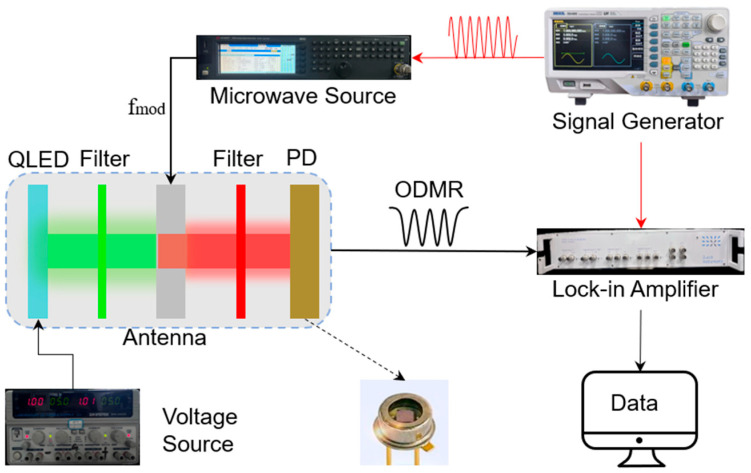
QLED-NV magnetometer array testing system.

Figure 2 shows the test system of the QLED-NV magnetometer array, where continuous-wave, optically detected magnetic resonance (CW-ODMR) was used for magnetic field detection. The magnetic sensing probe is powered using a GWINSTEK GPS-4303C voltage source from GWINSTEK Electronics Co., Ltd. in Taiwan, China. The microwave signal source is provided by a Keysight N5181B signal generator from Keysight Technologies (China) Co., Ltd (Xi’an, China). For sensitivity testing, a RIGOL DG4202 signal generator from RIGOL Technology Co., Ltd (Shenzhen, China) is used to provide a sine-wave signal, while modulation and demodulation functions are achieved using a HF2LI lock-in amplifier from Techu Scientific (Tianjin) Co., Ltd. (Tianjin, China).

## 3. Results and Discussion

### 3.1. Design and Characterization of Magnetometer Array

**Figure 3:**
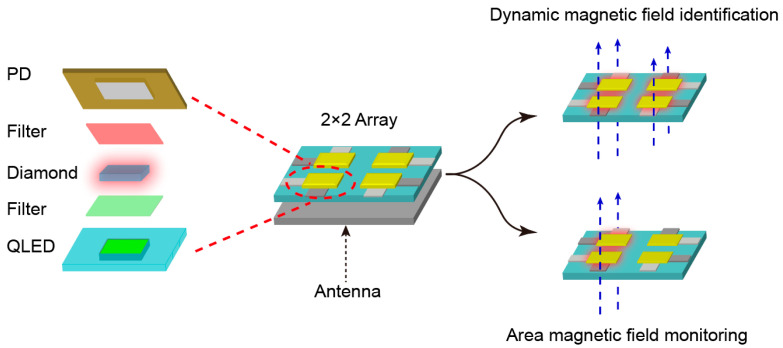
The 2 × 2 magnetometer array structure and its detection of regional magnetic field and dynamic magnetic field.

The individual unit structure of the 2 × 2 QLED-NV magnetometer array is composed of a QLED, a narrowband filter (532 nm), a diamond substrate, an antenna, and a bandpass filter (600–800 nm). To ensure uniform microwave driving across all units, we employed a pre-existing large-area antenna positioned beneath the array. The full design is schematically shown in Figure 3.

**Figure 4:**
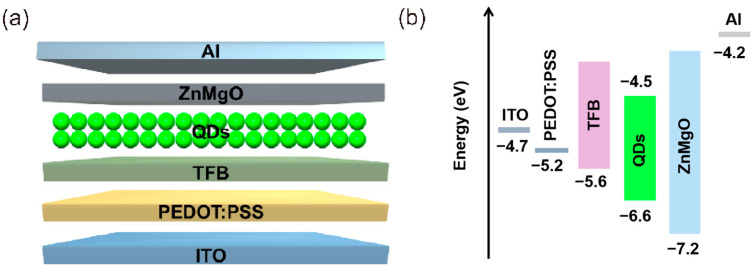
QLED device structure. (**a**) Thin-film structure of QLED device. (**b**) Energy-band diagram of QLED device.

The QLED device employs a CdSe/ZnS core-shell quantum-dot emission layer, with a multilayered structure composed of the following components (Figure 4a): a patterned indium tin oxide (ITO) glass substrate, a poly(3,4-ethyleneedioxyth-iophene):polystyrene sulfonate (PEDOT:PSS, 45 nm) hole injection layer, a poly(9,9-dioctylfluorene-co-N-(4-(3-meth-ylpropyl))diphenylamine (TFB, 40 nm) hole transport layer, a CdSe/ZnS quantum-dot active layer (20 nm), a zinc magnesium oxide (ZnMgO, 60 nm) electron transport layer, and an aluminum cathode (Al, 100 nm). The corresponding energy-band alignment is illustrated in Figure 4b. Prior characterization of these QLEDs demonstrated an electroluminescence (EL) peak at 532 nm with a narrow full width at half maximum (FWHM) of 20 nm. The QLED light-emitting layer (532 nm) is precisely matched with the energy level of the NV center (Figure 4b). Its emission wavelength (532 nm) is determined by the physical properties of the materials constituting the QLED itself and is not affected by fluctuations in operating voltage. Its narrow peak emission (FWHM = 20 nm) ensures efficient excitation of the NV center, and the microwave antenna (2.87 GHz) synchronously controls the electron spin state, forming a closed-loop sensing chain of “light excitation, microwave regulation and fluorescence reading”. The luminous intensity of a QLED is positively correlated with the driving voltage. A higher driving voltage injects more electron–hole pairs, which recombine and emit light in the QD layer. When operated at a 5 V bias, the device achieves a luminance of 10^5^ cd m^−^^2^, which is sufficient to effectively excite NV centers [31], yielding a fluorescence signal intensity of 50 mV. Crucially, this operating voltage does not induce structural degradation, ensuring stable device performance under continuous operation.

**Figure 5:**
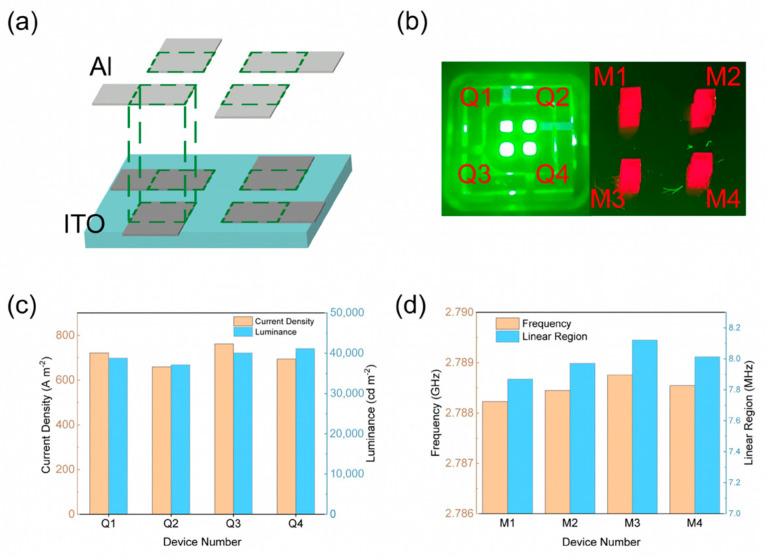
The 2 × 2 magnetometer array and its performance: (**a**) controlling for the luminous area of the QLED; (**b**) numbering QLED array and magnetometer array; (**c**) performance comparison of QLEDs prepared in the same batch; (**d**) resonant frequency and linear range of four units (M1 to M4).

To meet the excitation requirements of the 2 × 2 QLED-NV magnetometer array, we designed patterned ITO anodes and Al cathodes using photolithographic masking techniques to adjust the QLED emission area. When a driving voltage is applied to the electrodes of a QLED, electrons and holes are injected from the cathode and anode, respectively, and recombine in the quantum-dot (QD) layer, causing the quantum dots to radiate and emit light. As shown in Figure 5a, the emission area of each of the four units is 1 × 1 mm^2^, and each emission unit can be individually controlled. The emission effect of the 2 × 2 QLED array is displayed in the left panel of Figure 5b [32,33]. The fabricated QLED array units were labeled Q1, Q2, Q3, and Q4, and their consistency was tested under a 5 V applied voltage. Figure 5c shows that the current density of the four devices ranges from 660 A m^−2^ to 760 A m^−2^, while the luminance ranges from 38,000 cd m^−2^ to 42,000 cd m^−2^. These variations may result from minor thickness differences in the internal thin-film layers of the devices.

**Figure 6:**
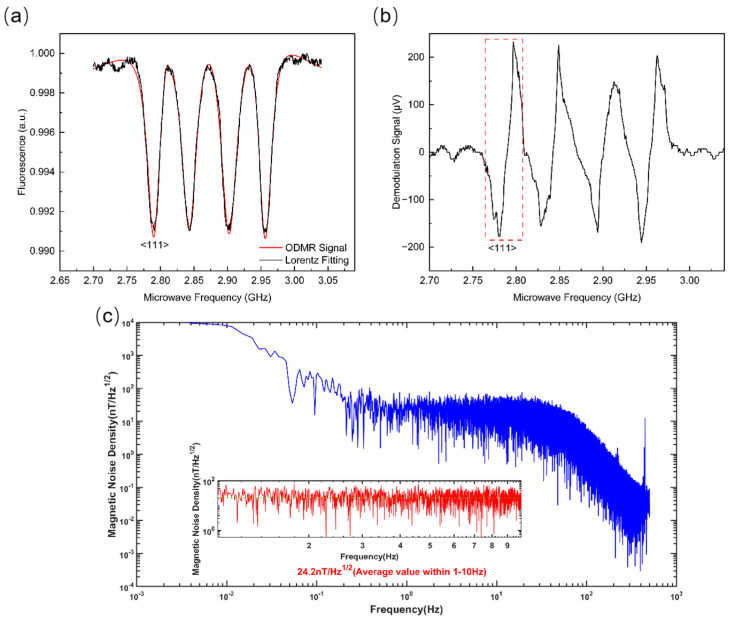
Performance of NV center magnetometer unit M1. (**a**) Normalized ODMR spectrum, where the two outermost peaks correspond to the <111> axis. (**b**) The demodulation curve corresponding to Figure 5a, where the red box corresponds to a single peak on the <111> axis. (**c**) Magnetic noise power spectral density.

To verify whether these discrepancies impact the excitation of NV centers, we constructed NV magnetometers above four QLEDs, labeled M1, M2, M3, and M4 (Figure 5b, right panel), to evaluate their respective performance. The ground state of a diamond NV color center is a triplet state consisting of three spin-energy levels (m_s_ = −1, 0, +1). In the absence of a magnetic field, m_s_ = ±1 is in a degenerate state, representing a single energy level. When an external-bias magnetic field is applied, the electrons in the NV color center’s ms = ±1 state undergo Zeeman splitting, lifting the degenerate state. The magnitude of the splitting is proportional to the magnetic field’s strength ($\gamma_{e}$ = 28 MHz/mT). When reflected on the ODMR signal, the wave trough of ODMR will undergo splitting, resulting in a frequency shift of Δ*f*. NV centers in diamond exhibit four distinct crystallographic orientations. When an external magnetic field is applied, its projections onto these NV axes vary, inducing splitting in the ODMR signals and thereby compromising measurement accuracy. To enhance precision, single-axis ODMR signals must be isolated. We selected <111>-axis NV center diamond as the magnetometer core and applied a bias magnetic field along the <111> axis. The QLED-NV magnetometer array is fixed above the optical platform using an optical post, while the magnet is mounted on a three-axis translation stage and placed beneath the device. By adjusting the translation stage, a magnetic field parallel to the <111> axis of the NV centers in the diamond within the integrated device can be applied. Under this bias-field configuration, NV centers aligned with the <111> crystallographic axis experience maximal magnetic influence, resulting in the greatest ODMR signal shift along this orientation [34,35,36,37]. This phenomenon manifests as the outermost single-peak resonance in the composite ODMR spectrum. This occurs when the applied external-bias magnetic field parallel to the <111> axis is B = 3 mT, which manifests as the splitting of two valleys into four valleys on the ODMR spectrum, i.e., the outermost two peaks corresponding to the <111> axis are completely separated from the peaks in other directions. For subsequent testing, the leftmost ODMR resonance peak post splitting was selected as the magnetometer output. Magnetic field detection was performed via continuous-wave, optically detected magnetic resonance (CW-ODMR), integrated with lock-in detection [38,39,40] to enhance system stability and the signal-to-noise ratio. Experimental parameters included a bias field of B = 3 mT, with signal generator outputting a sine reference modulation frequency with an amplitude of 1 V and a modulation frequency of 500 Hz. Figure 6a shows the ODMR curve of unit M1 after applying a bias magnetic field. Signal occupancy can lead to a decrease in sensitivity. To avoid excessive signal broadening, we used lower microwave power, ultimately resulting in a decrease in ODMR signal contrast. The contrast of the single peak on the left side of ODMR is 0.72%, which is sufficient for subsequent performance testing of magnetic sensing probes. Therefore, we chose to select the leftmost single peak for subsequent calculations. In Figure 2, we use the same channel of the signal generator to generate two sine-wave signals with exactly the same amplitude and frequency (with amplitude set to 1 V and a modulation frequency of 500 Hz), one of which is used as the modulation signal input for the microwave source and the other of which is connected to a lock-in amplifier as the reference signal required for demodulation. The operating frequency of the microwave source is precisely fixed at the single-peak valley position of the NV color center along the <111> crystal direction. Subsequently, the modulated signal is superimposed with a fixed-frequency microwave signal to generate a modulated microwave signal. Based on the characteristic of an ODMR signal changing with microwave frequency, the modulated microwave signal is applied to diamond using the sweep frequency method. After being treated by diamond, it is finally converted into amplitude modulation of the ODMR spectral signal. Finally, the modulated ODMR signal is filtered and demodulated using a lock-in amplifier to obtain the demodulation curve shown in Figure 6b.

Primary determination of measurement ranges is required for the four magnetometer units. Due to inherent unit-to-unit variations among the magnetometers, their respective demodulation curves exhibit non-uniform linear regimes. Experimental characterization revealed resonance frequencies spanning 2.7882 GHz to 2.7887 GHz across the four units. Demodulation of ODMR signals yielded linear-regime bandwidths ranging from 7.86 MHz to 8.12 MHz (see Figure 5d). The demodulation curve in Figure 6b indicates the linear interval where the output signal is proportional to the change in microwave frequency, marked within the red dashed box. To ensure synchronized operation within linear regimes across the magnetometer array, we harmonized the effective measurement ranges of all units. The measurable range was calculated using Equation (1):

(1) $$\Delta B=\frac{\Delta f}{\gamma_{e}}$$

In the equation, Δ*f* denotes the linear-regime bandwidth, with *γ_e_* = 28 MHz/mT representing the gyromagnetic ratio. The finalized fixed microwave resonance frequency was determined as 2.788 GHz, establishing an effective measurement range of 240 μT (from 120 to 120 μT) for the magnetometer array.

Magnetic noise spectral density serves as the critical parameter characterizing the power distribution of ambient magnetic field fluctuations across frequencies, which directly determines the sensitivity and operational performance of quantum sensors. The mathematical formulation is expressed as follows:

(2) $$\eta =\frac{ASD}{k\cdot \gamma_{e}}$$

**Figure 7:**
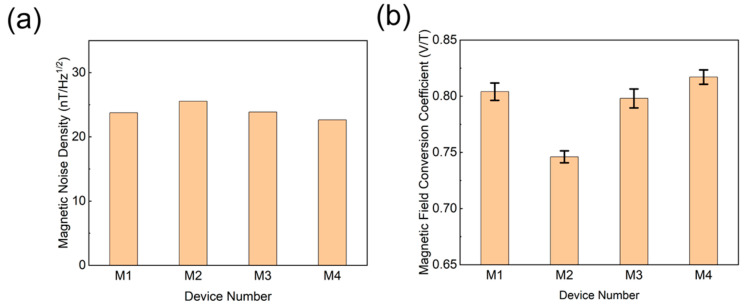
(**a**) Comparison of magnetic noise spectral density of each unit in magnetometer array. (**b**) The correlation curve between output voltage and applied magnetic field.

The demodulation curve slopes of magnetometer units M1–M4 were calibrated as k = 0.0286 mV/MHz, 0.0265 mV/MHz, 0.0285 mV/MHz, and 0.0289 mV/MHz, respectively. In this study, k represents the slope of the fitted curve at the zero point of the demodulation curve, $\gamma_{e}$ is the spin ratio of 28 MHz/mT, and ASD is the amplitude spectral density of the system noise. We used a lock-in amplifier to collect system background noise for 1 h without signal input. By performing a Fourier transform on the acquired signal, the magnetic noise spectral density curve over the full frequency range is obtained. The region below 1 Hz is usually affected by 1/f noise, while the effective signal amplitude decreases sharply above 10 Hz. Therefore, manually selecting the 1–10 Hz range can better reflect the practical sensitivity of near-DC magnetic field measurements [41]. After filtering of the entire system, the effective noise range is finally adjusted to 1–10 Hz (as shown in Figure 6c). We convert the measured background noise into ASD and substitute it into Formula (2) for calculation. The magnetic noise spectral density of unit M1 is approximately η ≈ 24.2 nT·Hz^−^^1/2^. As illustrated in Figure 7a, the measured sensitivity ranged from 22.8 nT·Hz^−^^1/2^ to 25.6 nT·Hz^−^^1/2^ across the four units. These variations primarily stem from positional inaccuracies in bias-field alignment and residual field inhomogeneities, with deviations remaining within permissible experimental error margins. The results confirm strong functional consistency among the magnetometers and demonstrate negligible performance degradation of NV diamond sensors caused by minor QLED fabrication discrepancies.

Due to inherent performance variations among the four magnetometer units (M1–M4) arising from fabrication tolerances, their responsiveness to magnetic field perturbations differs systematically. These discrepancies directly modulate the efficacy of magnetic-to-electrical signal transduction, necessitating individualized calculation of the magnetic field conversion coefficient for each unit. The theoretical magnetic field conversion coefficient is defined as follows:

(3) $$V=k\cdot \gamma_{e}\cdot B$$

In the equation, *γ_e_* = 28 MHz/mT represents the gyromagnetic ratio and k denotes the slope of the demodulation curve. Substituting the demodulation curve slopes (k) of the four magnetometer units into Equation (3), the theoretical magnetic-to-electrical conversion coefficients for M1 to M4 were calculated as 0.801 V/T, 0.752 V/T, 0.798 V/T, and 0.809 V/T, respectively. To validate the deviations between experimental and theoretical magnetic-to-electrical conversion coefficients, we measured voltage outputs under varying applied magnetic fields with a fixed bias field of B = 3 mT. During testing, the demodulation curve was locked at its zero point using a lock-in amplifier [42]. The applied field was systematically swept from −120 μT to +120 μT in 20 μT increments, with 20 repeated measurements per field step.

The experimental magnetic field conversion coefficients for M1–M4 were determined as (0.804 ± 0.0077) V/T, (0.746 ± 0.0053) V/T, (0.798 ± 0.0082) V/T, and (0.817 ± 0.0064) V/T, respectively (see Figure 7b). Comparative analysis between these experimental values and their theoretical counterparts revealed minor discrepancies, with all units exhibiting maximum relative errors below 3%—well within permissible experimental tolerances. This agreement underscores the high reliability of the theoretical model and validates the robustness of the experimental methodology. The strong consistency observed in the conversion coefficients across the magnetometer array further confirms the system’s operational stability and measurement reproducibility. The difference in magneto-electric conversion coefficients between the various units in this experiment is mainly due to the uneven spin-coating thickness caused by the QLED electron transport layer (ZnMgO) spin-coating process, which leads to uneven excitation of the luminescent layer. In the future, improvements will be made in the process. These results provide critical empirical support for the refinement of sensor design and the advancement and practical deployment of NV-diamond magnetometers in high-precision magnetic sensing applications.

### 3.2. Detection of Regional Magnetic Field

**Figure 8:**
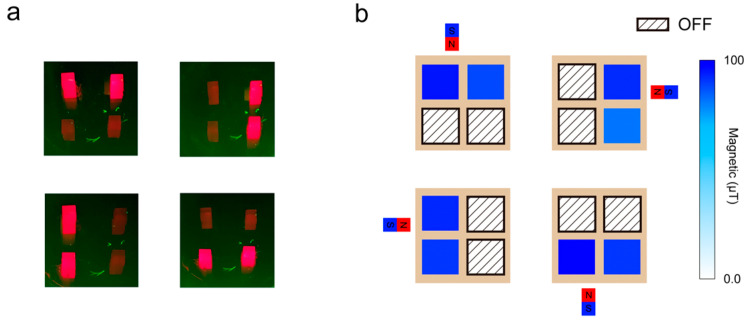
Detection of regional magnetic field. (**a**) Turn on magnetometer units in different areas. (**b**) Test the magnetometer’s ability to identify regional magnetic fields.

To further validate the operational feasibility of the NV magnetometer array in practical scenarios, we conducted rigorous testing of its spatial field discrimination capability. The array’s performance in identifying localized magnetic fields was methodically evaluated under varying regional field conditions [43]. During experimentation, two individual units on the collateral side of the QLED-NV magnetometer array were selectively activated (Figure 8a) to emulate distinct operational states. When an external magnetic field was applied, we intentionally biased the field toward one unit while monitoring the array’s real-time detection response. The maximum applied field intensity was set to B = 100 μT to assess detection limits under high-field conditions.

As demonstrated in Figure 8b, biasing the magnetometer toward one side resulted in a marked enhancement of the local magnetic field intensity on that side, confirming the array’s ability to selectively amplify detection sensitivity in regions aligned with external field gradients. This intrinsic adaptability enables the NV diamond magnetometer array to achieve precise spatial discrimination of magnetic fields under diverse environmental conditions.

Further analysis of the experimental data reveals that monitoring inter-unit differential values not only enables robust detection of magnetic field presence but also facilitates preliminary localization of field sources. These results conclusively demonstrate that the NV diamond magnetometer array can selectively activate region-specific detection units in complex field environments while leveraging inter-unit signal disparities to achieve effective spatial mapping of magnetic anomalies.

### 3.3. Detection of Dynamic Magnetic Field

**Figure 9:**
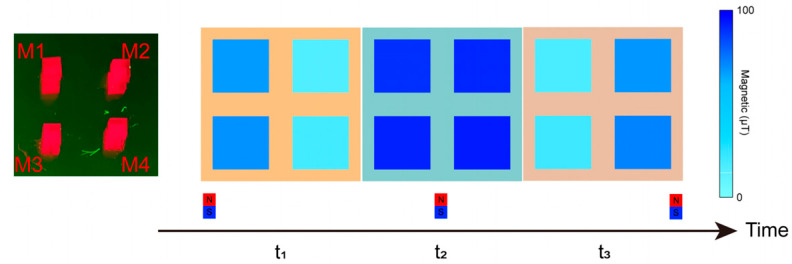
Dynamic magnetic field identification. The left picture shows a schematic diagram of all four units being turned on. The right picture shows the detection of dynamic magnetic field by the magnetometer array. At time t_1_, the magnetic field is located on the left side of the magnetometer array; at t_2_, the magnetic field is located below the magnetometer array; at time t3, the magnetic field is located on the right side of the magnetometer array.

Following the validation of spatial field discrimination capabilities, we simultaneously activated all four units of the device to evaluate its performance in detecting time-varying magnetic fields. To ensure experimental accuracy, the QLED-NV magnetometer array was rigidly mounted, while an external field source was translated along a predefined linear trajectory from left to right. Throughout the process, a high-speed data acquisition system recorded time-resolved magnetic field magnitudes from all four units, corresponding to three critical source positions: left-aligned, center-aligned (directly below the array), and right-aligned, as shown in Figure 9.

During the experiment, at time t_1_, when the magnetic field was positioned to the left of the magnetometer array, units M1 and M3—proximate to the field source—recorded elevated magnetic flux densities of 69.37 μT and 63.22 μT, respectively. In contrast, M2 and M4 registered significantly lower values of 19.48 μT and 15.71 μT, indicating strong field localization on the left side and validating the proximity-dependent sensitivity of the array.

At t_2_, as the field source transitioned to the center-aligned position directly beneath the array, all four units exhibited uniformly high field strengths: M1 and M3 measured 92.64 μT and 89.65 μT, while M2 and M4 detected 93.55 μT and 91.16 μT, respectively. This symmetric response confirmed the array’s ability to resolve centrally located fields with balanced sensitivity across all units.

Finally, at t_3_, with the field shifted to the right side, M2 and M4 displayed markedly increased readings of 75.67 μT and 69.56 μT, whereas M1 and M3 decreased to 21.63 μT and 17.57 μT. The rate of change in magnetic field detection is mainly determined by the movement speed of the external magnetic field source and the non-radiative relaxation of the diamond NV color center. These results conclusively demonstrate the array’s capacity to track dynamic field displacements spatially and quantify positional changes through differential unit responses.

**Figure 10:**
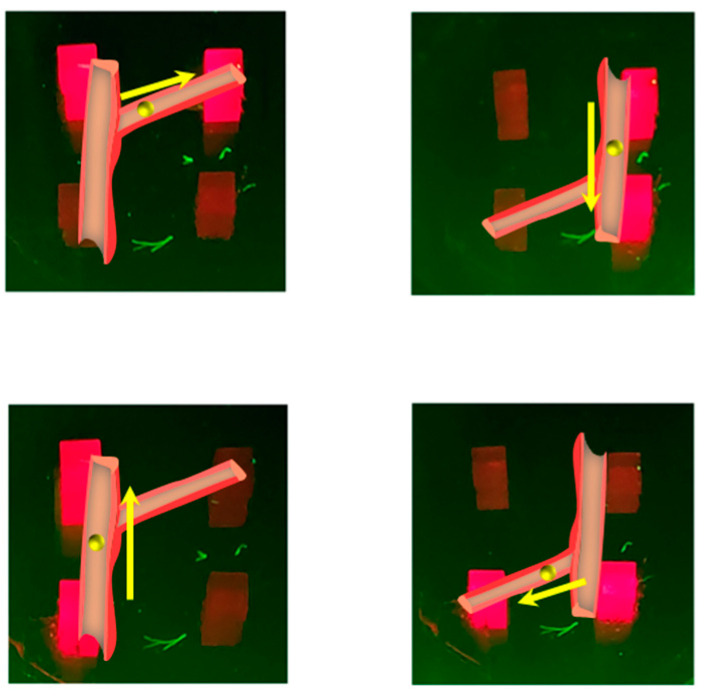
Motion trajectory monitoring of magnetic robots in a simulated vascular environment.

To demonstrate the practical application value of the QLED-NV sensor array in real-world scenarios, we simulated and demonstrated the area localization function of a magnetic robot flowing in blood vessels (Figure 10). When the magnetic robot moves in a simulated vascular environment, the sensor array can recognize its location in real time: when the magnetic robot flows in the main blood vessel and approaches the left units (M1 and M3), the QLED unit in the corresponding area is activated. When the magnetic robot enters the branch blood vessels (approaching M1 and M2), the signal in the original area is automatically turned off, and the QLED unit in the corresponding area is activated. This “regional response feedback” mechanism reflects the ability of sensor arrays to detect dynamic magnetic fields, providing feasible technical solutions for precise tracking and non-invasive monitoring of magnetic carriers in the biomedical field.

Analysis of the experimental data reveals that the QLED-NV magnetometer array can dynamically identify the spatial localization and intensity distribution of magnetic fields during external field variations. This capability not only demonstrates the array’s high efficiency and accuracy in real-time dynamic field detection but also provides critical insights for further device performance optimization. Through this experiment, we validated the array’s core functionality: its adaptability to region-specific magnetic field distributions and its ability to selectively activate specific pixel elements in targeted regions, thereby significantly reducing overall power consumption. This design feature enhances its operational flexibility and energy efficiency in practical applications.

Moreover, the array’s regionalized detection capability enables real-time tracking of field dynamics and rapid identification of potential magnetic anomalies. Such a technological breakthrough paves the way for transformative applications in geophysical exploration, biomedical imaging, and intelligent robotic navigation, establishing a robust foundation for next-generation magnetic sensing technologies.

## 4. Conclusions

In this study, leveraging the controllable emissive area characteristic of QLED technology, we propose a monolithically integrated arrayed design for NV center-based magnetometers. To preliminarily validate this approach, a 2 × 2 QLED-NV magnetometer array prototype was fabricated. Specifically, four independent emissive zones were patterned on a single QLED substrate, each integrated with a dedicated magnetometer unit. The array achieved a magnetic sensitivity of 26 nT/√Hz and an effective measurable range of ±120 μT. Following uniformity testing across the four units, simulated experiments further confirmed the array’s ability to detect region-specific static fields and dynamic magnetic variations.

This work establishes a novel methodological framework for future large-scale integrated magnetometer arrays. The experiment not only validates the core functional merits of the QLED-NV array but also highlights its significant potential in practical applications, such as high-resolution field mapping and real-time anomaly detection. Compared with the traditional 532 nm laser (with a volume of about 142.5 × 60 × 50 mm and a cost of about USD 3660), the single QLED light source proposed in this study has a volume of only about 1.5 mm×1.5 mm×200 μm and a single-chip cost of less than USD 10. As a pioneering advancement, this innovation offers underpinning and technical benchmarks to drive scalable quantum magnetometer arrays toward higher integration, sensitivity, and adaptability.

## Data Availability

The original contributions presented in this study are included in the article. Further inquiries can be directed to the corresponding author.
